# Supplementary material for: Development of chemokine network inhibitors using combinatorial saturation mutagenesis
Source: Commun Biol. 2025 Apr 3;8:549. doi: 10.1038/s42003-025-07778-6 (PMC11969024; doi:10.1038/s42003-025-07778-6)
Supplement: Supplementary file 4 — Supplementary Data 2 [file 42003_2025_7778_MOESM4_ESM.zip › SupplementaryData3/scripts/xattune_dr.tar!/./script/analysis/JK/JK_E163_CXCL9_ATC_plot.pdf]

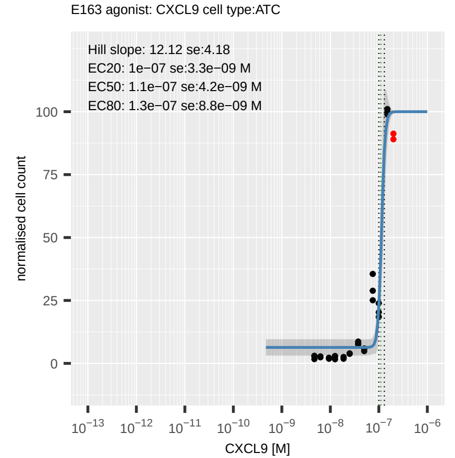
E163 agonist: CXCL9 cell type:ATC
Hill slope: 12.12 se:4.18
EC20: 1e−07 se:3.3e−09 M
EC50: 1.1e−07 se:4.2e−09 M
EC80: 1.3e−07 se:8.8e−09 M
normalised cell count
100
75
50
25
0
$10^{-13}$
$10^{-12}$
$10^{-11}$
$10^{-10}$
$10^{-9}$
$10^{-8}$
$10^{-7}$
$10^{-6}$
CXCL9 [M]
